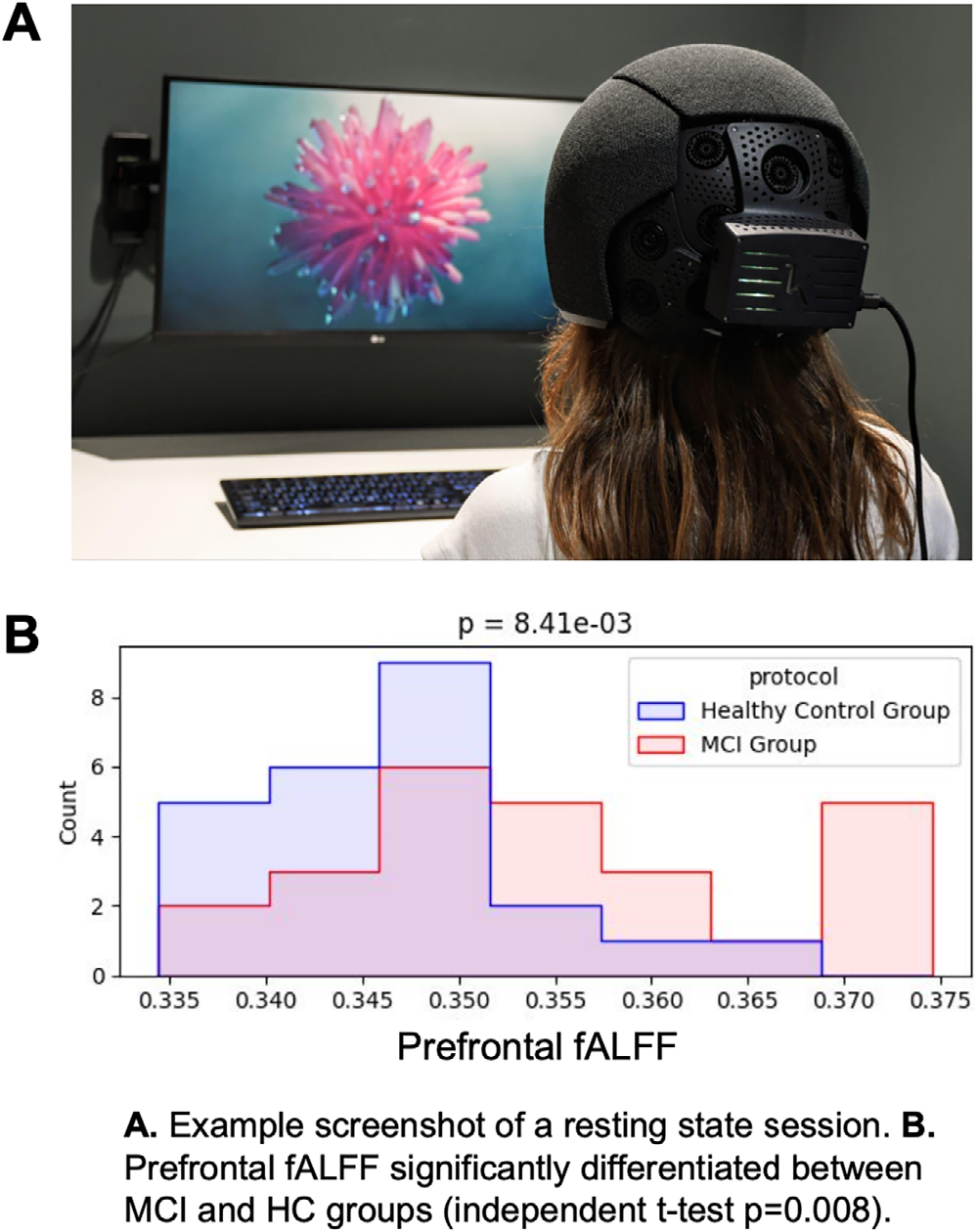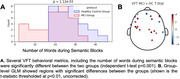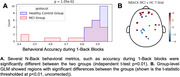# Developing a novel brain‐based biomarker of mild cognitive impairment using time‐domain functional near‐infrared spectroscopy

**DOI:** 10.1002/alz.093856

**Published:** 2025-01-09

**Authors:** Julien Dubois, Ryan M Field, Sami Jawhar, Erin M Koch, Zahra M. Aghajan, Naomi Miller, Katherine L Perdue, Moriah Taylor

**Affiliations:** ^1^ Kernel, Los Angeles, CA USA

## Abstract

**Background:**

Mild cognitive impairment (MCI) is largely under‐diagnosed by primary care physicians. There is an urgent need to develop new objective screening tools to assist with early detection of MCI. Time‐domain functional near‐infrared spectroscopy (TD‐fNIRS) can be used to measure brain function in clinical settings and may fill this need.

**Method:**

MCI patients (n=21) and control older adults (HC; n=24) participated in brain recordings using TD‐fNIRS during rest and cognitive tasks implicated in MCI: 1) Verbal fluency task (VFT) consisting of interleaved blocks reciting words (control: weekdays; semantic: words from a given category; phonological: words starting with a given letter); 2) N‐back task targeting working memory by testing memory for patterns with specific memory loads (0‐, 1‐ and 2‐backs) in a block‐design manner. Raw data, i.e. the distributions of the times of flight (DToFs) of photons at two wavelengths (690 and 905nm), underwent standard preprocessing to obtain moments of DToFs and corresponding changes in the concentrations of oxygenated/deoxygenated hemoglobin. Features related to brain function, systemic physiology, and head optical properties were extracted. For tasks, behavioral metrics were also computed.

**Result:**

First, we looked for features that significantly differentiated between MCI and HC groups during a resting state session (watching a 7‐minute audiovisual segment; Figure 1A). Multiple features, including head optical properties and prefrontal fractional Amplitude of Low Frequency Fluctuations (fALFF) were statistically different between MCI and HC (Figure 1B). Next, we explored the tasks both in terms of behavior and neural features. In VFT, behavior was significantly different between groups (Fig. 2A), which is expected from prior literature. Additionally, whole‐brain hemodynamic activation maps, as computed using Generalized Linear Models (GLM) revealed areas with significant differences between the two groups (Fig. 2B). Similarly, for the N‐back task, multiple behavioral metrics were separated between the groups (Fig. 3A) and group‐level GLM showed brain regions with significantly different activations between groups, specifically medial prefrontal cortex (Fig. 3B).

**Conclusion:**

We demonstrated significant group‐level differences between MCI vs healthy controls using TD‐fNIRS. As such, with ongoing data collection, we seek to validate these findings and employ classification techniques for MCI detection.